# Insulin enables acquisition of the IL7R^+^ memory phenotype in PD1^+^ T cells in RA tissues

**DOI:** 10.1038/s41419-026-08916-6

**Published:** 2026-05-25

**Authors:** Venkataragavan Chandrasekaran, Malin C. Erlandsson, David Svensson, Eric Malmhäll-Bah, Sofia Töyrä Silfverswärd, Rille Pullerits, Gergely Katona, Maria I. Bokarewa

**Affiliations:** 1 Department of Rheumatology and Inflammation Research, Institute of Medicine, University of Gothenburg, Gothenburg, Sweden; 2 Rheumatology Clinic, Sahlgrenska University Hospital, Gothenburg, Region Västra Götaland Sweden; 3 Department of Clinical Immunology and Transfusion Medicine, Sahlgrenska University Hospital, Gothenburg, Region Västra Götaland Sweden; 4 Department of Chemistry and Molecular Biology, Science for Life Laboratory, Faculty of Science and Technology, University of Gothenburg, Gothenburg, Sweden; 5 Department of Medical Biochemistry and Cell Biology, Institute of Biomedicine, University of Gothenburg, Gothenburg, Sweden

**Keywords:** Autoimmunity, Insulin signalling, Transcriptomics

## Abstract

Insulin signaling regulates cellular metabolism in an epigenetic manner, but its role in the immune cell homeostasis remains unknown. High plasma insulin obstructs efficient insulin signaling and rewires metabolic activity in autoimmunity. In this study, we explored the functional consequences of insulin signaling for the metabolism and phenotype of effector CD4^+^ T cells in blood and synovial tissue of patients with rheumatoid arthritis (RA). Transcriptome profiling of CD4^+^ cells in RA blood and synovia revealed high metabolic activity and effector function of the survivin/*BIRC5*^hi^PD1^hi^ T peripheral helper cell population. Low insulin signaling and deficient histone acetylation in RA T cells amplified proinflammatory IFNγ and TNF expression. Co-deposition of survivin with acetylated histone H3K27 on regulatory chromatin controlled the transcription of histone acetylation complex subunits and insulin-dependent genes. Insulin stimulation and histone deacetylase inhibition induced an increase in histone acetylation. In CD4^+^ cell cultures and in aggressive PD1^hi^Tph cells in RA synovial tissue, exposure to insulin synergized with inhibition of histone deacetylation to upregulate IL7 production suppressing IFNγ and PD1. This activated IL7R-signaling mediators STAT5A/B, BCL2, and promoted acquisition of CD27^+^CD45RO^+^ central memory phenotype in the PD1^hi^Tph cells. Likewise, the CD4^+^ cells in hyperinsulinemic T2D patients showed enrichment of IL7R^+^T cell cluster. In RA patients, antagonizing folate transport and JAK/STAT signaling activated insulin signaling and histone acetylation-dependent metabolism of CD4^+^ cells. Concomitant with CTLA4-dependent signaling, this enabled the adoption of an incipient IL7R^+^ T cell phenotype. This study demonstrates that insulin binds together metabolic activity and histone acetylation in CD4^+^ cells. Sufficient insulin signaling promotes IL7R^+^ memory phenotype accrual in aggressive PD1^hi^Tph cells. Hence, achieving insulin sensitivity via histone acetylation disarms effector CD4^+^ T cell function and presents an attractive interventional goal to restore immune cell homeostasis in RA.

## Introduction

Insulin is broadly appreciated as a hormone that regulates the extracellular levels of glucose. However, insulin signaling has several other roles in non-metabolic tissues. A recent view of the insulin signaling network proposed its functional metabolic, mitogenic and cytoskeletal remodeling arms in brain tissue, liver, and kidneys [1]. Cells of the adaptive immune system were notably absent, highlighting that research on insulin signaling in this context remains insufficient.

The effector activity of T cells is energy-intensive and fuels IFNγ production. In energy-demanding states, cells utilize aerobic glycolysis not to maximize ATP yield, but to sustain metabolic flux, redox balance, and anabolic metabolism [2–5], while the pentose phosphate pathway is engaged in supporting redox balance and nucleotide biosynthesis. This form of adaptation is prevalent in autoimmune rheumatoid arthritis (RA) [3, 6, 7] and in type 2 diabetes (T2D) [8, 9], conditions wherein the balanced flow of glucose utilization is affected via altered insulin signaling, leading to chronic inflammation. Reduced insulin sensitivity is an emerging characteristic feature in RA patients [10, 11], whose pathogenic T cells are amenable to targeting by anti-rheumatic drugs [3, 12]. Aside from altered insulin signaling, both RA and hyperinsulinemic T2D share common features such as the influence of systemic inflammation associated with increase in levels of TNF and IFNγ, genetic predisposition, and changed gut microbiota composition, all of which contribute to the expansion of pathogenic T cell phenotypes. This makes the understanding of insulin signaling in T cells central for RA pathogenesis. In this regard, epigenetic modifications are an important factor coordinating insulin signaling and directly influencing the gene transcription of proinflammatory cytokines and enzymes of different metabolic pathways.

Maintaining T cell homeostasis is facilitated by epigenetic modifications of histones, DNA methylation and adequate levels of metabolites [13]. Modification of histones by attaching methyl or acetyl groups to specific residues of histone proteins, is important for T cell differentiation and function. For example, the MLL1 lysine methyltransferase catalyses lysine-4 trimethylation (H3K4) and remodels chromatin accessibility aiding T cell protection against autoimmunity [14]. Similarly, EZH2 which catalyses trimethylation of lysine-27 on the histone H3 tail (H3K27), stabilizes expression of the key transcription factors *Tbx21* and *Gata3* thereby controlling optimal Th1 and Th2 cellular responses [15]. The balance between histone H3K27 and H3K4 trimethylation orchestrates DNA damage response in IFNγ-producing CD4^+^T cells [16, 17].

Histone H3 acetylation onsets gene transcription by relaxing the condensed chromatin structure. Acetyl Co-A derived from pyruvate decarboxylation is used by histone acetyltransferases (HAT) to perform this function, which is reversed by histone deacetylases (HDAC). Blocking pyruvate decarboxylation affects T cell function by reducing acetyl Co-A availability and limiting histone acetylation [18]. Metabolic activity and change in T cell function often depend on levels of different metabolites. For example, high lactate levels activated the lactate dehydrogenase enzyme and increased the regulatory phenotype of CD4^+^T cells thereby promoting immunosuppression [19]. Glucose-derived pyruvate chromatin remodeling in CD4^+^T cells coupled activity of pyruvate dehydrogenase enzyme to histone H3K27 acetylation [2]. These studies emphasize why glycolytic metabolism is important for T cell functions, highlighting the need to understand how insulin signaling modulates these processes in autoimmune inflammation.

In this study, we investigated the metabolism and phenotype of pathogenic CD4^+^T cells in RA tissue and their response to insulin signaling. We found that insulin signaling defines metabolically active and proinflammatory PD1^hi^CD4^+^T cells in RA blood and synovial tissue. Co-deposition of survivin-H3K27ac on regulatory chromatin controls transcription of the HAT/HDAC enzymes responsive to insulin. Insulin signaling remarkably increased H3K27ac deposition and promoted transition of PD1^hi^Tph cells to IL7R^+^ memory T cells. Finally, by screening modern immunosuppressive drugs we explored the molecular pathways for their ability to change insulin effects in RA T cells.

## Results

### Histone acetylation and insulin signaling enhances metabolic activity of CD4^+^ cells

**Fig. 1:**
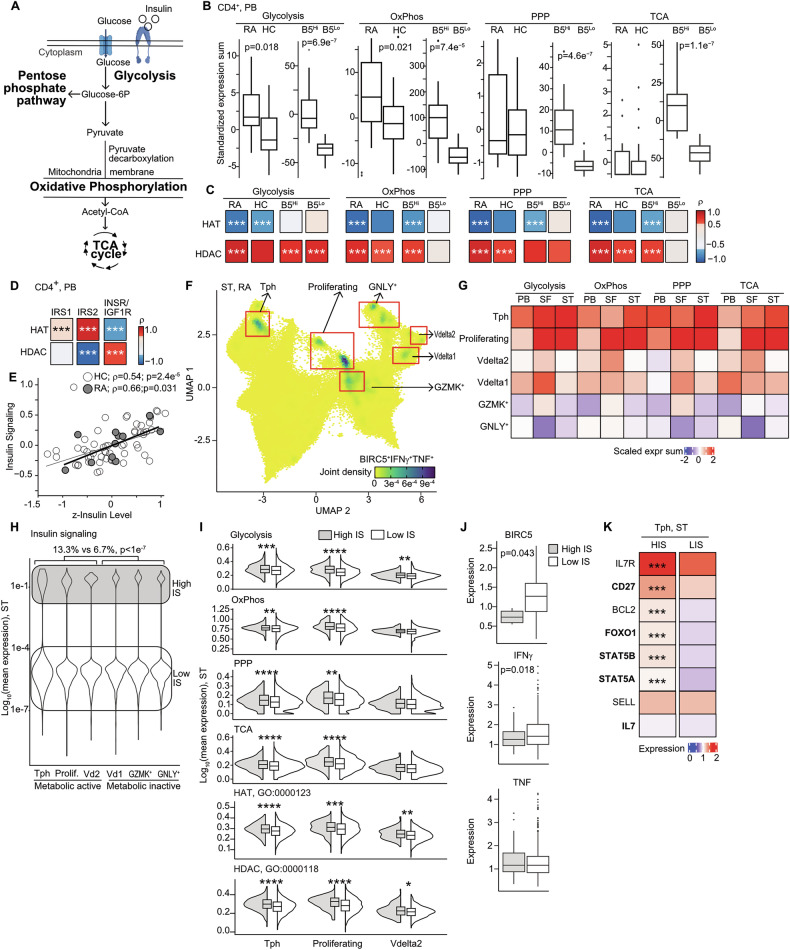
BIRC5^hi^CD4^+^ cells represent active insulin-sensitive clusters in RA tissues. **A** Glucose metabolism. Studied metabolic pathways are in bold. **B** Box plots of standardized expression sum (SES), by RNA-seq, of genes within metabolic pathways in CD4^+^ cells of RA (*n* = 11), healthy controls (HC, *n* = 57), and of CD4^+^ cells split by mean expression of *BIRC5* gene in high (B5^hi^) and low (B5^lo^) (*n* = 24). Boxes indicate IQR and whiskers indicate the minimum and maximum values. GO-terms used for pathway annotation: Glycolysis (GO:0006096); oxidative phosphorylation (OxPhos, GO:0006119 and GO:0022900); pentose phosphate pathway (PPP, GO:0006098); tricarboxylic acid cycle (TCA, GO:0006099). *P*-values are calculated by Wilcoxon unpaired test. **C** Heatmap of Spearman’s correlation between HAT, HDAC and metabolic pathways as above. GO-terms used for annotation HAT (GO:0000123) and HDAC (GO:0000118). Asterisks indicate *p*-values. * < 0.05, ** < 0.01, *** <0.001. **D** Heatmap of Spearman’s correlation between HAT and HDAC and insulin signaling (IS). Asterisks indicate *p*-values * < 0.05, ** < 0.01, *** <0.001. **E** Scatter plot of Spearman’s correlation between plasma insulin levels and SES of insulin signaling. **F** Uniform manifold approximation and projection (UMAP) map of scaled expression intensity of *BIRC5*, *IFNG*, and *TNF* in T cells of RA synovial tissue (ST), by single cell transcriptome. *BIRC5*^*Hi*^CD4^+^ clusters are indicated. **G** Heatmap of scaled expression intensity of metabolic pathway (annotated as above) in peripheral blood (PB), synovial fluid (SF), and ST of RA patients, by scRNA-seq. **H** Violin plot of insulin signaling by SES of *INSR, IRS1, IRS2 and IGF1R* genes in *BIRC5*^Hi^CD4^+^ clusters. P-value was calculated by chi-square test. **I** Violin plot of metabolic pathways SES in cells with high and low IS in *BIRC5*^*Hi*^CD4^+^ clusters. *P*-values are calculated by Wilcoxon unpaired test. Asterisks indicate * < 0.05, ** < 0.01, *** <0.001, **** <0.0001 **J**. Box plot of *BIRC5, IFNG*, and *TNF* expression in Tph cells with high and low IS. Boxes indicate IQR and whiskers indicate the minimum and maximum values. *P*-values are calculated by Wilcoxon unpaired test. **K** Heatmap of expression for IL7R-signaling targets in Tph cells high and low IS. Genes regulated by survivin-H3K27ac are in bold. *P*-values are calculated by Wilcoxon unpaired test. Asterisks indicate * < 0.05, ** < 0.01, *** <0.001, **** <0.0001.

We investigated the metabolic profile of CD4^+^ cells by studying expression of genes involved in the canonical metabolic pathways (Fig. 1A) in RA patients and healthy controls (HC) (Supplementary Table ST1). The normalized gene expression in the glucose metabolism pathways in CD4^+^ cells of 11 treatment naïve RA patients and 57 HC showed activation of glycolysis, and oxidative phosphorylation pathways, while pentose phosphate pathway, and tricarboxylic acid cycle were similar to HC (Fig. 1B). High expression of *BIRC5* was associated with upregulated transcription of the metabolic pathways in CD4^+^ cells (Fig. 1B).

Glucose metabolism via insulin signaling is regulated by the balance between HAT and HDAC [20]. Analysis of the correlation between HAT and HDAC enzymes and metabolic profile of CD4^+^ cells revealed that expression of HDAC enzymes was positively correlated to the metabolic profile in CD4^+^ cells of both RA and HC (Fig. 1C). On the other hand, HAT expression was inversely related to the metabolic pathways in CD4^+^ cells, and it was specifically pronounced in *BIRC5*^hi^CD4^+^ cells.

Next, we assessed the association between histone acetylation and the insulin signaling mediators (ISM) defined by *INSR, IGF1R, IRS1* and *IRS2* genes. Expression of ISM was strongly correlated to the plasma insulin levels (Fig. 1E) reflecting insulin sensitivity. The expression of *IRS1* and *IRS2* was positively correlated to HAT expression, while the *INSR* to *IGF1R* expression ratio was negatively correlated to HAT (Fig. 1D), replicating its inverse relationship to the metabolic profile of CD4^+^ cells (Fig. 1C).

Together, the findings demonstrated that insulin signaling and enhanced metabolic activity of CD4^+^ cells was tightly linked to histone acetylation and high *BIRC5* expression.

### High insulin signaling marks aggressive Tph cluster in RA synovia

These findings urged us to investigate whether metabolic activity demarcated specific CD4^+^T cells that contributed to chronic inflammation in RA. Markers of the 24T cell clusters were mapped to the single-cell transcriptome of CD4^+^ cells in blood and synovial fluid of 8 RA patients and extended further into synovial tissue (ST) of 82 RA patients with active disease.

The combined expression of *BIRC5*, *IFNG*, and *TNF* was highest in the clusters of PDCD1^+^CXCL13^+^ Tph cells, PTTG1^+^TUBA1B^+^ proliferating cells, CD4^+^GZMB^+^CD45RO^–^GNLY^+^ cells, GZMK^+^ cells, Vdelta1 and Vdelta2 cells (Fig. 1F, Supplementary Fig. S1A). The aggressive nature of these cell clusters was revealed by a strong correlation between the metabolic pathway genes and the cluster markers (Supplementary Table ST2). The cluster markers were also significantly upregulated in blood *BIRC5*^hi^CD4^+^ cells of RA patients (Supplementary Fig. S1B). The survey of these six clusters demonstrated that Tph, proliferating, and Vdelta2 cells were metabolically active across blood, synovial fluid, and ST (Fig. 1G, Supplementary Fig. S1C), and were characterized by high transcription of the enzymes mediating the canonical metabolic pathways.

Assessing insulin sensitivity in the metabolically active T cell clusters, we found that Tph, proliferating, and Vdelta2 cell clusters had a significantly increased prevalence of ISM compared to the other clusters (13.3% vs 6.7%, *p* < 1e−7) (Fig. 1H). The metabolic activity and HAT-HDAC expression were significantly upregulated in Tph and proliferating cells having high ISM (Fig. 1I), while expression of *BIRC5, IFNG, TNF* in these cells was lower (Fig. 1J). In contrast, metabolic activity was largely independent of ISM in the remaining ST clusters (Supplementary Fig. S1D, E).

In summary, the single-cell transcriptome analysis revealed differential metabolic and HAT/HDAC activity among the CD4^+^T cells of RA-ST. The active metabolic profile and high expression of HAT/HDACs in Tph, and proliferating clusters were dependent on insulin signaling.

### Insulin increases histone acetylation by controlling HAT and HDAC complexes via chromatin co-deposition of survivin and H3K27ac

**Fig. 2:**
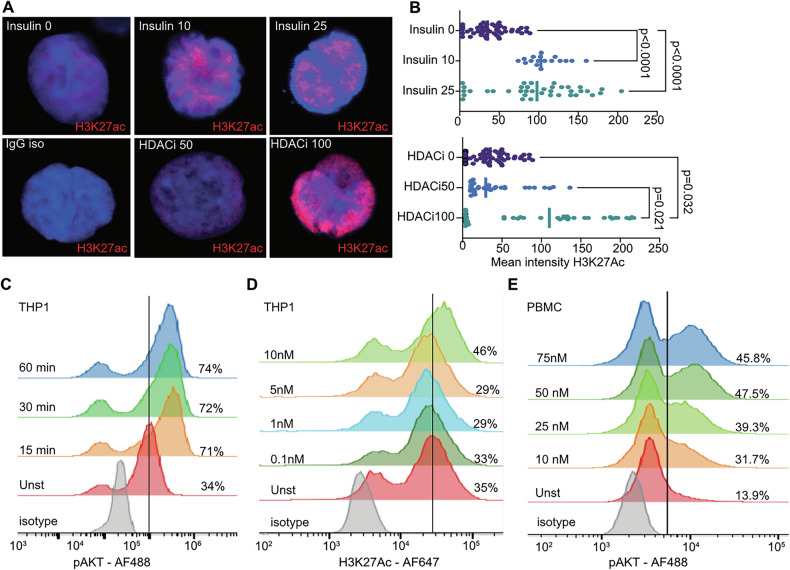
Insulin signaling induces phosphorylation of AKT1 and increases histone acetylation. **A** Confocal microscopy images of THP1 cells stained with antibodies to H3K27ac (red) in nucleus (blue) after stimulation with insulin (top), and HDAC-inhibitor (bottom) for 24 h. Images are acquired with 40× magnification with additional digital magnification 1.5×. **B** Scatter plot of nuclear H3K27ac staining intensity after stimulation with insulin (top) and HDAC-inhibitor (bottom). *P*-values are calculated by Wilcoxon unpaired test. Asterisks indicate * < 0.05, ** < 0.01, *** <0.001, **** <0.0001. **C** Histogram of phosphorylated serine 473 (p)AKT1 mean fluorescence intensity in THP1 cells stimulated with 10 nM insulin, by flow cytometry. **D** Histogram of H3K27ac mean fluorescence intensity in THP1 cells stimulated with increasing concentrations of insulin for 24 h, by flow cytometry. **E** Histogram of pAKT1 mean fluorescence intensity in human lymphocytes stimulated with increasing concentrations of insulin for 30 min, by flow cytometry.

Next, we investigated the intracellular effects of insulin and histone deacetylation in culture. Using confocal microscopy and flow cytometry, we found that both insulin and HDACi increased H3K27ac deposition in nuclei of THP1 cells (Fig. 2A, B). Quantifying H3K27ac deposition through mean fluorescence intensity, we demonstrated that insulin dose-dependently increased the nuclear H3K27ac staining (Fig. 2B, D). Further, THP1 cells treated with insulin had a linear increase in the prevalence of H3K27ac-positive foci per cell (Supplementary Fig. S2A).

To validate insulin dependence of histone acetylation, we measured phosphorylation of AKT1 at the serine residue 473 as an early event of the receptor tyrosine kinase signaling activation (Fig. 2C). Insulin treatment induced an increase of pAKT signal from 34% in unstimulated THP1 cells to 71% in insulin-stimulated cells. The pAKT increase occurred within the first 15 min and was maintained at that level for at least 60 min. The parallel THP1 cultures were stained for H3K27ac after incubation with insulin for 40 h (Fig. 2D). In these cells, H3K27ac signal increased from 35% to 46%. In the insulin-treated PBMC cultures gated on lymphocytes and single cells using the forward scatter and side scatter plots, we found accumulation of pAKT signal within the first 30 min of insulin exposure, which showed an obvious 2.28 times enrichment with pAKT positive cells in the cultures treated with 10 nM insulin and further enrichment to 3.42 times in the cultures treated with 50 nM insulin (Fig. 2E).

**Fig. 3:**
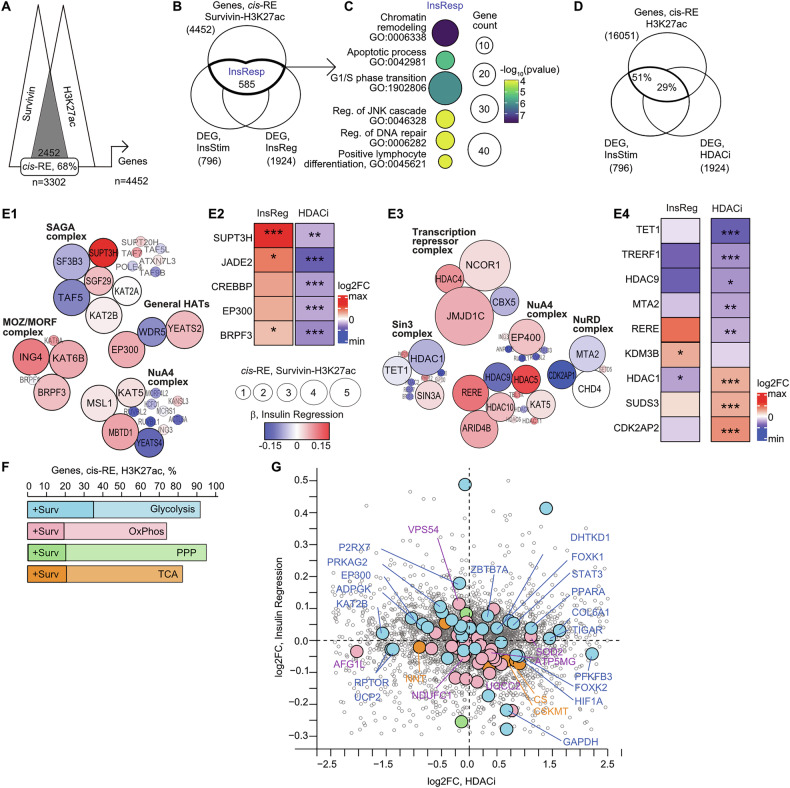
Co-deposition of survivin-H3K27ac on chromatin controls transcription of the acetylation complex subunits. **A** Analysis strategy of survivin and H3K27ac deposition, by ChIP-seq, in cis-regulatory elements (*cis*-RE) and connected genes in human CD4^+^ T cells. **B** Venn diagram of insulin-responsive (InsResp) genes connected to *cis*-RE with survivin-H3K27ac co-deposition. Genes are identified by DESeq2 analysis of CD4^+^ cells transcriptome after a) direct insulin stimulation (InsStim) and b) in regression to corresponding plasma insulin levels, by RNA-seq. **C** Scatter plot of pathway enrichment of insulin-responsive genes, by GSEA GO-terms. Circle size indicates the number of genes in the pathway, and color intensity indicates *p*-value. **D** Venn diagram of insulin-responsive (InsResp) genes connected to *cis*-RE with H3K27ac deposition. Genes are identified by DESeq2 analysis of CD4^+^ cells transcriptome after a) direct insulin stimulation (InsStim) and b) HDAC inhibition. **E** Scatter plot of protein subunits with survivin-H3K27ac in HAT (E1) and HDAC (E3) complexes, annotated by STRING database. Circle size represents number of *cis-*RE with survivin-H3K27ac connected to the gene. Circle color represents beta-coefficient of expression difference by DESeq2 analysis of CD4^+^ cell transcriptome in regression to corresponding plasma insulin levels. (E2 and E4) Heatmap of transcription difference, by log2FoldChange (FC), in CD4^+^ cells in regression to plasma insulin levels (InsReg), and after HDAC inhibitor treatment in vitro. **F**. Bar plot of percentage of H3K27ac and survivin-H3K27ac connected genes in each metabolic pathway. GO-terms used for pathway annotation as in Fig. 1. **G** Scatter plot of expression difference of survivin-H3K27ac connected genes in transcriptome of CD4^+^ cells in regression to plasma insulin levels and after HDACi treatment, by RNA-seq. Expression difference is calculated by DESeq2 analysis. Colour fill corresponds to metabolic pathway annotation of the gene.

Next, we investigated if insulin effects were functionally dependent of H3K27 acetylation. To assess this, we performed chromatin immunoprecipitation in primary CD4^+^T cells using antibodies targeting survivin and H3K27ac and sequenced the antibody-bound chromatin regions. Computational overlap of survivin and H3K27ac co-deposition identified these proteins in 2452 regions across the human genome. More than one-third of these regions were situated within *cis*-regulatory elements (*cis*-RE) (Fig. 3A). Annotating the *cis*-RE to the confirmed connected genes [21], we identified 4452 protein-coding genes potentially dependent on the survivin-H3K27ac co-deposition in CD4^+^T cells. Next, we experimentally identified 585 insulin-responsive genes by combining the differentially expressed genes in insulin-stimulated CD4^+^T cell cultures (Supplementary Table ST3) and the insulin-responsive genes identified by regression analysis of CD4^+^ cells transcriptome to plasma insulin levels (Fig. 3B, Supplementary Table ST4). Functional enrichment analysis of the insulin-sensitive genes revealed chromatin remodeling to be the topmost enriched pathway, followed by apoptosis, and cell cycle progress (Fig. 3C, Supplementary Tables ST5, ST6). Further, 80% of the DEG in insulin-stimulated CD4^+^T cell cultures were regulated by *cis-RE* containing deposition of H3K27ac, 30% of which were also sensitive to HDAC inhibition (Fig. 3D).

Encouraged by the observation that insulin regulates chromatin remodeling via survivin-H3K27ac co-deposition, we explored if HAT/HDAC complexes were affected by insulin stimulation. To do this, we extracted the protein subunits annotated to the HAT/HDAC complexes and searched them among the survivin-H3K27ac-connected genes. We found that the genes of multiple subunits in HAT and HDAC complexes were regulated by >1 *cis*-RE containing survivin-H3K27ac co-deposition (Fig. 3E1, E3). Moreover, linear regression analysis of the CD4^+^T cells transcriptome to the plasma insulin levels showed that, in HAT complexes, increasing insulin levels impacted expression of scaffolding subunits *BRPF3* in HBO1/KAT7 complex, *JADE2* of the MOZ/MORF complex, and *EP300*, and *CREBBP* subunits in general H3-HAT complex (Fig. 3E2). In HDAC complexes, the survivin-H3K27ac co-deposition regulated the transcription repressor complex via *HDAC4*, Sin3 complex via *HDAC1*, and the NuRD complex via *MTA2* (Fig. 3E3). Interestingly, exposure to insulin significantly upregulated the HAT subunits *BRPF3, EP300, CREBBP, JADE2* which were downregulated by HDACi, demonstrating concordant effect of insulin and histone deacetylation (Fig. 3E2). In contrast, HDAC enzymes *HDAC9, HDAC10*, and *TET1* sensitive to HDACi remained unaltered by insulin (Fig. 3E4).

A majority of the genes involved in metabolic pathways were enriched in genes controlled by H3K27ac deposition (Fig. 3F). Among these, 17-36% of the genes involved in glucose utilization were supervised by at least one *cis*-RE containing survivin-H3K27ac (Fig. 3F). The strongest effect of insulin and HDACi was found in the glycolytic genes *EP300, KAT2B, PFKFB3, UCP2, TIGAR, GAPDH, HIF1A, STAT3, RPTOR, FOXK2, COL6A1, PPARA*, *ADPGK, DHTKD1, PRKAG2, FOXK1, P2RX7, ZBTB7A*, closely followed by the OxPhos genes *NDUFC1, AFG1L, ATP5MG, UQCC2, VPS54*, while PPP and TCA were largely unaffected (Fig. 3G).

Together, the analysis of chromatin deposition revealed that insulin induced an increase in histone acetylation by controlling transcription of the HAT/HDAC complexes.

### Insulin promotes transition of effector PD1^+^T cells into the central memory IL7R^+^T cells

**Fig. 4:**
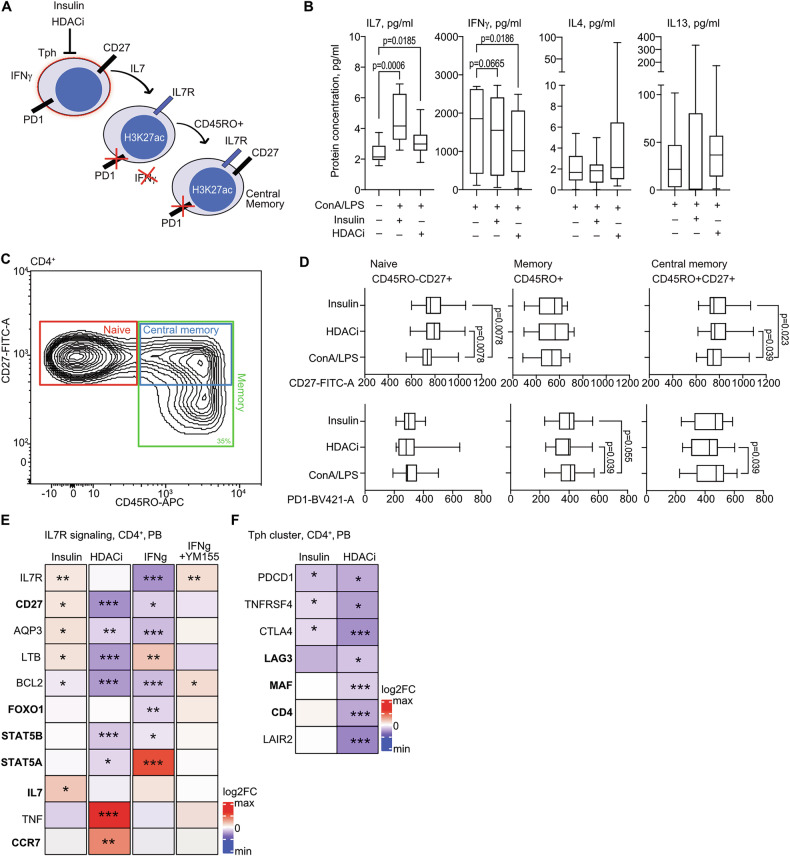
Insulin and HDAC inhibition promote transition of PD1^hi^ Tph cells to IL7R^+^ memory T cells. **A** Transition model of PD1^hi^ Tph cells to the IL7R^+^ T memory cells, influenced by insulin and HDAC inhibition. **B** Box plot of protein cytokine levels in supernatants of CD4^+^ cells (*n* = 12) stimulated with insulin (25 nM) and HDACi (50 µg/mL) for 24 h, by ELISA. Boxes indicate IQR and whiskers indicate the minimum and maximum values. *P*-values are calculated by Wilcoxon Sign rank test. **C** Gating strategy to CD4^+^ T cell subset analysis by flow cytometry. **D** Box plot of PD1 and CD27 expression in CD4^+^ cells (*n* = 8) stimulated by insulin and HDACi, by mean fluorescence intensity (MFI). Boxes indicate IQR and whiskers indicate the minimum and maximum values. *P*-values are calculated by Wilcoxon Sign rank paired test. **E** Heatmap of expression difference of IL7R-related signaling molecules in CD4^+^ cells after in vitro stimulation with insulin (25 nM), HDACi (50 µg/mL), IFNγ (50 ng/ml), and survivin inhibitor YM155 (10 μM), by RNA-seq. Genes connected to *cis*-RE containing survivin-H3K27ac co-deposition are indicated bold. Asterisks indicate RNA-seq *p*-values * < 0.05, ** < 0.01, *** <0.001, **** <0.0001. **F** Heatmap showing expression difference, by log2FC, of cluster markers of Tph cluster after insulin stimulation, HDAC inhibition. In bold are the genes supervised by a cis-RE containing survivin-H3K27ac deposition. *P*-values are calculated by DESeq2 analysis. Asterisks indicate nominal *p*-values * < 0.05, ** < 0.01, *** <0.001, **** <0.0001.

Since the aggregated findings in blood and synovial tissue CD4^+^ cells (Fig. 1) and our experimental results (Fig. 2) demonstrated that high insulin signaling was prevalent in active PD1^+^Tph clusters, we sought to replicate these findings by investigating effect of insulin and HDACi on the T cell phenotype (Fig. 4A).

We found that both insulin and HDACi increased production of IL7 protein by the cultured cells, the cytokine important for survival and proliferation of memory T cells [22] (Fig. 4B). In contrast, production of the T_h_1 master cytokine IFNγ was suppressed by insulin and HDACi. The T_h_2 cytokines IL4 and IL13 were not affected by insulin or HDACi, pointing out that the effector T cells producing IFNγ were the primary targets of this stimulation.

To investigate immediate effect of insulin and HDACi on T cell development, we performed phenotyping of cultured CD4^+^ cells by flow cytometry. Analyzing the surface expression of CD27 and PD1 receptors in naïve CD45RO^–^CD27^+^, memory CD45RO^+^, and central memory CD45RO^+^CD27^+^ T cell subsets (Fig. 4C), we found that both insulin and HDACi significantly increased expression of CD27 in naïve and central memory CD4^+^ cells, advancing the IL7-sensitive memory phenotype (Fig. 4D). Simultaneously, insulin and HDACi suppressed expression of PD1 in the central memory CD4^+^ cells, aiding reduced T cell activation justified by low IFNγ production by those cells (Fig. 4D).

Next, we evaluated the relationship between IFNγ-dependent Tph phenotype and IL7R signaling using transcriptome of CD4^+^ cells. We found that insulin stimulation and survivin inhibition by YM155 upregulated *IL7R*, CD27, and *BCL2*, the key signaling molecules downstream of IL7R, which were also markedly downregulated by HDACi and IFNγ stimulation (Fig. 4E). The *CD27, FOXO1, STAT5A* and *STAT5B* genes downstream of IL7R possessed at least one *cis*-RE containing survivin-H3K27ac co-deposition highlighting the connection between histone acetylation, survivin, and sufficient insulin signaling. Consequently, the Tph cluster markers *PDCD1*, *TNFRSF4*, *CTLA4*, and *LAG3* were downregulated by insulin and HDACi (Fig. 4F). Of those, only *LAG3* had survivin-H3K27ac co-deposition. To explore the insulin-dependent changes in RA-ST, we asked if high insulin signaling assisted acquisition of the IL7R^+^ phenotype in the metabolic active CD4^+^ clusters. In agreement with experimental findings, the high ISM in Tph cluster of RA-ST coexisted with increased IL7R^+^ signaling (Fig. 1K).

Together, our findings convincingly demonstrated that sufficient insulin signaling and HDACi induced IL7 and suppressed IFNγ production, which concomitantly promoted the transition of the aggressive PD1^+^Tph cell phenotype to the memory phenotype of IL7R^+^T cells in blood and ST.

### IL7R^+^ clusters are prevalent in hyperinsulinemic T2D, augmented by biguanide treatment

**Fig. 5:**
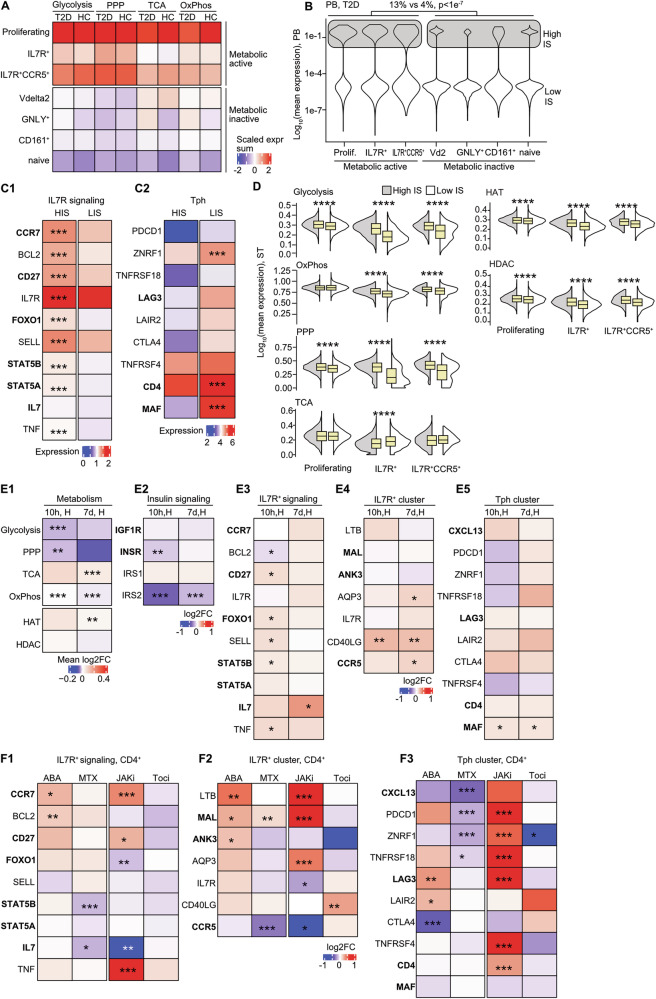
Expansion of IL7R^+^ memory cells in the metabolic active CD4^+^ cells of hyperinsulinemic patients with type 2 diabetes. **A** Heatmap of standardized expression sum (SES) of metabolic pathway genes in CD4^+^ T cell clusters of type 2 diabetes (T2D) and healthy controls (HC), by single cell transcriptome. GO-terms used for pathway annotation are Glycolysis (GO:0006096); oxidative phosphorylation (OxPhos, GO:0006119 and GO:0022900); pentose phosphate pathway (PPP, GO:0006098); tricarboxylic acid cycle (TCA, GO:0006099). **B** Violin plot of insulin signaling (IS) SES of genes *INSR, IRS1, IRS2, IGF1R* in T cell clusters of T2D and HC. Chi-square test *p*-value is indicated. **C** Heatmap of expression for IL7R-signaling targets (C1) and markers of Tph cluster (C2) in IL7R^+^ clusters cells with high and low IS. *P*-values are calculated by Wilcoxon unpaired test. Asterisks indicate *p*-values * < 0.05, ** < 0.01, *** <0.001, **** <0.0001. **D** Violin plot of SES of metabolic pathways (annotated as above) in metabolic active cells with high and low IS of T2D patients. *P*-values are calculated by Wilcoxon unpaired test. Asterisks indicate *p*-values * < 0.05, ** < 0.01, *** <0.001, **** <0.0001. **E** Heatmap of expression difference in metabolic pathways (E1), insulin signaling (E2), IL7R-signaling targets (E3), markers of IL7R^+^ cluster (E4) and Tph clusters (E5) in RNA-seq PBMC of HC orally treated with metformin for 10 h, 7 days. Difference is calculated by Deseq2 analysis and presented by log2 fold change (FC). Asterisks indicate nominal *p*-values, *<0.05, ** < 0.01, *** < 0.0001. **F** Heatmap of expression difference in IL7R-signaling targets (F1), markers of IL7R^+^ clusters (F2) and Tph cluster (F3), by RNA-seq of CD4^+^ T cells from RA patients treated with JAKi (25 treated, 9 untreated), abatacept (ABA1, *n* = 22, paired), methotrexate (MTX, *n* = 28, paired), tocilizumab (Toci, *n* = 6, paired). Difference is calculated by Deseq2 analysis and presented by log2FC. Asterisks indicate *p*-values * < 0.05, ** < 0.01, *** <0.001, **** <0.0001.

To explore relevance of the discovered insulin signaling for transition to IL7R^+^ cells in non-inflammatory environment, we investigated the single-cell transcriptome of 31,777 CD4^+^T cells from 7 T2D patients with hyperinsulinemia and 3 healthy controls [23], asking whether hyperinsulinemia was associated with the effects observed in cultured cells exposed to insulin. Mapping the markers of the 24T cell clusters (Supplementary Fig. S3A), we found that blood T cells of T2D patients were different from those in RA-ST. Specifically, the cells comprised only seven CD4^+^T cell clusters with a cell count >1% (Fig. 5A, Supplementary Fig. S3B, Supplementary Table ST7). In hyperinsulinemic T2D and in HC, the proliferating cell cluster and the two IL7R^+^ clusters had high transcription of the canonical metabolic pathways, while the Vdelta2, GNLY^+^, CD161^+^ and naïve T cell clusters were metabolic dormant.

Similar to the RA-ST, we separated the cells with high and low insulin signaling based on the mean expression sum of ISM genes in each cluster (Fig. 5B). The high ISM in T2D CD4^+^ cells significantly upregulated the metabolic pathways and HAT/HDAC enzymes in the IL7R^+^ and proliferating clusters (Fig. 5D). Like in RA-ST, we found that the metabolic active proliferating and IL7R^+^T clusters had higher prevalence of high ISM cells (13% vs 4%, *p* < 1e−7). Target genes downstream of IL7R, including *CD27, BCL2, CCR7, STAT5A* and *STAT5B* were significantly overexpressed in metabolically active cells with high ISM (Fig. 5C1). Consequently, markers of Tph cells start emerging in the cells with low ISM (Fig. 5C2). These cells had significantly higher expression of IFNγ and TNF (Supplementary Fig. S3E).

To investigate further functional causality between cellular insulin sensitivity and propagation of IL7R^+^ clusters, we used the blood leukocyte transcriptome of HC prepared from paired samples collected before and after oral use of biguanide [24], that improve cellular insulin sensitivity by activating AMPK [25, 26]. We found a downregulation of ISM molecules *INSR* and *IRS2* after 10 h biguanide treatment (Fig. 5E2). The downregulation of insulin signaling was followed by suppression of glycolysis, PPP, and OxPhos, and upregulation of TCA and HAT (Fig. 5E1). Concomitantly, biguanide aided early upregulation of genes downstream of IL7R (Fig. 5E3), which was later accompanied by upregulation of *IL7* and the IL7R^+^ cluster markers *CD40LG* and *CCR5*. The Tph markers remained largely unaffected by biguanide with the exception for *MAF* (Fig. 5E5).

Together, single-cell transcriptome of T2D CD4^+^ cells revealed abundance of insulin signaling in the metabolic active T cells. Similar to RA ST, high insulin signaling was associated with enrichment for IL7R^+^T cells. Activation of AMPK promoted further the IL7R^+^T cell enrichment.

### Molecular mechanisms counteracting insulin signaling

Aiming to understand which pro-inflammatory pathways prevented sufficient insulin signaling in RA, we explored the publicly available transcriptome of CD4^+^ cells of RA patients treated with inflammation targeting drugs including paired samples prepared before and after treatment with the folate inhibitor methotrexate [27], CTLA4 co-stimulatory inhibitor abatacept [28], and the IL6 receptor inhibitor tocilizumab [29] (Supplementary Table ST1). The effect of inhibitors of Janus-kinase (JAKi) downstream mediators of IL7R, was studied in a cross-sectional comparison of RA patients treated with JAKi and RA patients having no disease-modifying drugs [12].

We found that the *CCR7, BCL2, CD27, FOXO1* genes downstream of IL7R, were all significantly upregulated in CD4^+^ cells after abatacept treatment (Fig. 5F1), which reproduced the direct effects of insulin stimulation (Fig. 4). Furthermore, the IL7R^+^ cluster markers were evidently upregulated with abatacept suggesting that CTLA4-dependent co-stimulation inhibition restricted expansion of IL7R^+^ clusters (Fig. 5F2). The support to IL7R^+^ cluster occurred despite that abatacept inhibited the genes of oxidative phosphorylation, required for memory T cell development, and had no effect on the ISM genes (Supplementary Fig. S3F, S3G). In contrast, methotrexate and JAKi upregulated the ISM genes contributing to activation of metabolic pathways and HAT/HDAC enzymes (Supplementary Fig. S3F, S3G). Also, MTX and JAKi enabled insulin responsiveness by inhibiting histone methylation genes *EZH2*, *KMT2A* and *PRKDC* in RA patients. Despite promoting insulin signaling and increased expression of the genes instrumental in the oxidative phosphorylation, both methotrexate and JAKi upregulated *MAL* and downregulated *IL7*, *IL7R* and the *CCR5*, *STAT5B* and *FOXO1* genes downstream of *IL7R* (Fig. 5F1, F2). JAKi mirrored insulin-dependent immune modulation by activating transcription of the memory genes *CD27*, *CCR7*, and *IL23A*. Methotrexate actively downregulated the Tph cell markers *CXCL13*, *PDCD1*, *ZNRF1*, and *TNFRSF18*, while JAKi upregulated these markers (Fig. 5F3). Tocilizumab suppressed transcription of glycolysis genes (Supplementary Fig. S3G). This had neither a measurable impact on the IL7R signaling nor on the markers of the Tph cluster.

Therefore, the analysis of CD4^+^ cell transcriptome revealed that targeted intervention of pro-inflammatory pathways of RA pathogenesis enabled acquisition of IL7R^+^ memory phenotype by activating T cell metabolism or inhibiting co-stimulation.

## Discussion

In this study, we demonstrate that high insulin signaling aids the metabolic activity of PD1^hi^CD4^+^ T cells in blood and ST of RA patients. The metabolic active T cells expressed survivin/*BIRC5*, *IFNγ*, and *TNF*, which supported their longevity and aggressiveness in RA. Epigenetic binding of survivin and H3K27ac within *cis-*RE controlled transcription of insulin-sensitive genes by affecting the HAT/HDAC complexes. The *BIRC5*^hi^CD4^+^ T cells in both blood and ST of RA patients were metabolically active and insulin sensitive. This harbours the dual opportunity of minimizing chronic inflammation by means of reducing IFNγ production and effector T cell function and, on the other side, maintaining immunological memory that fosters a threat of disease flare upon an occasional antigen challenge. Upon exposure to insulin, blood PD1^+^CD4^+^ T cells increased histone H3K27 acetylation and reduced IFNγ production to adopt a CD27^+^CD45RO^+^ memory phenotype. The expansion of IL7R^+^ memory T cells in PBMCs of hyperinsulinemic T2D patients suggest that these mechanisms exist in vivo, though T2D-specific factors such as systemic inflammation, metabolic status, sex hormone influence, treatment etc. may contribute to this expansion.

Blunted insulin signaling is positively associated with higher disease activity in RA [12] and is regarded a harbinger of poor treatment response [30]. Insulin stimulation affects T cell phenotype both directly via activation of the PI3-Akt pathway, and indirectly via activation of glycolysis and oxidative phosphorylation through increased histone acetylation. Memory T cells utilize oxidative phosphorylation [31–33] for their energy demands, a phenomenon which, together with the activated glycolysis, is demonstrated to be insulin dependent in this study. Limiting histone acetylation by reducing the pool of acetyl Co-A has been proposed as a solution to restrain the metabolism of effector CD4^+^ T cells in autoimmunity [5]. In addition, histone acetylation is essential for DNA damage repair at sites of histone H3 tail deposition [34] and drives T cell plasticity during antigen response [2, 5]. By studying the effects of inhibiting histone deacetylation in primary activated T cells, our study unifies these mechanisms of T cell homeostasis and emphasizes that histone acetylation bridges the insulin-dependent crosstalk between glucose metabolism and the effector-memory T cell function. Considering the broad nature of insulin-dependent effects on epigenetic processes controlling cell phenotype, one should undertake further perturbation experiments such as gene knockdown studies of the insulin receptor, receptor-ligand blocking through synthetic inhibitors, profiling chromatin accessibility after insulin/HDACi stimulation, and protein-protein interaction studies to establish direct causality and cell specificity of the presented findings.

Exposure of CD4^+^ cells to insulin suppressed transcription of immunological check-point receptors *PDCD1* and *CTLA4* and limited IFNγ production. The cytokine IL7 is instructive for the conversion, longevity, and proliferation of effector T cells to antigen-experienced memory T cells [22, 35]. In RA, this pro-survival cytokine can contribute to chronic inflammation by activating the effector CD4^+^T cells resident in the inflamed ST [36, 37]. Here, we highlight the insulin-sensitive nature of the PD1^hi^T and IL7R^+^T cells, which also possess features of the central memory phenotype by expressing *CD27, CCR7, STAT5A/B*, and *BCL2*. The counterbalancing nature of PD1 and IL7R axis was elucidated in experimental autoimmune diabetes, where IL7R blockade suppressed effector T cell responses by PD1 upregulation [9, 38]. The insulin-dependence of IL7R^+^ phenotype was also reinforced by analysis of CD4^+^ cells in T2D where high insulin signaling was connected to enhanced metabolic activity in IL7R^+^T cells.

Analyzing effect of modern anti-rheumatic drugs, we found that the transition of PD1^hi^Tph cells to IL7R^+^ central memory T cells required antagonism of folate transport and JAK/STAT signaling. Indeed, JAK/STAT inhibition activated the PD1^hi^Tph cluster, and modulated the IL7R^+^ cluster by enhancing ISM and glucose metabolism, upregulating *CCR7, CD27, MAL* and *AQP3*, and suppressing *IL7* and *IL7R*. In contrast, folate transport antagonism by methotrexate suppressed the gene markers of the PD1^hi^Tph clusters, while still activating ISM and the HAT complex. Notably, CTLA4-dependent control of CD4^+^ T cells in RA patients partially mimicked JAK/STAT inhibition effects by activating the IL7R^+^ cluster genes. However, the CTLA4-dependent effect on IL7R^+^ cells engaged neither the ISM, HAT/HDAC signaling nor the PD1^hi^Tph cluster genes, suggesting that this mechanism mediates accumulation of IL7R^+^ central memory cells by a mechanism independent on ISM and HDAC. Nevertheless, these findings raise interesting questions on the importance of the IL7R^+^ central memory T cell enrichment for success of the RA preventive interventional strategy [39]. Together, our drug response analysis illustrates the diversity of molecular mechanisms controlling pathogenic T cell behavior in RA.

Our study reveals that genes involved in cellular metabolism are epigenetically regulated by survivin and H3K27ac co-deposition on regulatory chromatin in CD4^+^ T cells. This finding agrees with several independent reports [3, 5, 16, 17, 34, 40, 41] that emphasize the importance of survivin and histone tail deposition in DNA damage repair, gene transcription, and metabolic adaptation. Enhancing on the previous reports, we demonstrate here that insulin responsiveness promotes histone acetylation through activation of HAT enzymes. The insulin-sensitive genes controlled by survivin-H3K27ac deposition were frequently annotated to the scaffolding subunits of the canonical HAT complexes, which were also sensitive to HDAC inhibition. Similar effect of survivin has been described for the assembly of BRG1/SWI complex that controls DNA accessibility [16], making this oncoprotein a key player in transcription factors scaffolding leading context-dependent epigenetic regulation of DNA remodeling, availability, and 3D organization. Taken together, the combination of experimental findings presented in this study provides strong rationale for the idea that promoting histone acetylation via increasing insulin sensitivity or HDAC inhibition in survivin-high proinflammatory CD4^+^ T cells can epigenetically reprogram metabolism and behavior of *BIRC5*^*hi*^Tph cells.

In conclusion, our study presents a molecular epigenomic mechanism of survivin-bound histone acetylation in asserting the insulin-sensitive IL7R^+^ central memory T cell phenotype in blood and synovial tissue of RA patients. We demonstrated that several pro-inflammatory pathways contributed to preventing the transition of PD1^hi^Tph cells to IL7R^+^ central memory T cells and could be targeted by immunological interventions. The clinical relevance of these discoveries for RA pathology calls for further investigation.

## Materials and methods

### Human material

This study used biological material collected from patients with rheumatoid arthritis (RA) and healthy controls summarized in Supplementary Table ST1. We used three independent patient cohorts, which were collected at the Rheumatology Clinic of Sahlgrenska University Hospital, Gothenburg, Sweden. Cohort 1 was collected between 10 January 2022, and 14 March 2023. It consists of 11 non-diabetic treatment naive RA patients and 57 non-diabetic subjects having no rheumatic diseases (healthy controls, HC), randomly selected among the 1st-visit patients with musculoskeletal complaints. Cohort 2 was randomly collected during November 2018 and consisted of 24 patients with established RA disease. Cohort 3 was collected between 7 October 2019 and 26 October 2020. It consists of 56 patients with established RA disease. All RA patients fulfilled the 2010 EULAR/ACR classification criteria [42]. Among the RA patients within cohort 3, 25 were treated with the Janus kinase inhibitors (JAKi). Twelve patients combined JAKi with methotrexate, four with other antirheumatic drugs, and one each with abatacept, tocilizumab, or sarilumab. Among the non-JAKi-treated patients, fourteen were treated with methotrexate monotherapy, ten with TNF inhibitors, and one with tocilizumab. Two of the JAKi-treated and three of the non-JAKi-treated patients used oral corticosteroids. Nine patients had neither methotrexate, JAKi, nor oral corticosteroids. None of the control subjects were using immune suppressive drugs or oral corticosteroids.

For the in vitro experiments and RNAseq of insulin-treated CD4+ cells blood of healthy non-diabetic subjects were used (16 female, 19 males; age 45.2 ± 11.5 years).

### Isolation and stimulation of cells

Human peripheral blood mononuclear cells (PBMC) were isolated from the venous heparinized peripheral blood by density gradient separation on Lymphoprep (Axis-Shield PoC As, Dundee, Scotland). PBMC cultures (1 ×10^6^ cells/mL) in RPMI medium (Gibco, Waltham, Massachusetts, USA) containing 50 μM β-mercaptoethanol (Gibco), Glutamax 2 mM (Gibco), gentamicin 50 μg/mL (Sanofi-Aventis) and 5% fetal bovine serum (Sigma-Aldrich) at 37 °C in a humidified 5% CO_2_ atmosphere. Cells were stimulated with concanavalin A (ConA, 0.625 μg/mL, MP Biomedicals), and lipopolysaccharide (LPS, 5 μg/mL, Sigma-Aldrich) for 48 h. For immunophenotyping, the cultures were supplemented with insulin (Humalog 100 U/mL, Eli Lilly, Indianapolis, IN, USA) 25 nM and/or the class I HDAC inhibitor (HDACi) valproate, 50 µg/mL (stock 200 mg/mL, Ergenyl, Sanofi, Paris, France) after 24 h. Supernatants were collected for cytokine measures and cells were analyzed by flow cytometry. For phospho-specific flow cytometry, PBMC cultures were treated with insulin (final concentration range 0–75 nM) for 30, 60 and 90 min and harvested by immediate fixation by addition of 4% formalin to stop dephosphorylation.

CD4^+^ cell cultures (1.25 × 10^6^ cells/mL) were prepared from fresh PBMC cultures by positive selection (Invitrogen, 11331D) and propagated as above. For ChIPseq analysis, CD4^+^ cells were stimulated with ConA and LPS as above for 24 h. For RNAseq and qPCR, cells were cultured in wells coated with anti-CD3 antibody (0.5 μg/mL; OKT3, Sigma-Aldrich, St. Louis, MO, USA), and treated with insulin (0 and 10 nM, Humalog, Eli Lilly) for 72 h, In separate experiments, CD4^+^ cells were stimulated with IFNγ (50 ng/ml; Peprotech, Cranbury, NJ, USA), and survivin inhibitor YM155 (46) (10 nM; Selleck Chemicals, Houston, TX) for 72 h.

### THP1 cell stimulation

Human monocytic cell line THP1 (TIB-202, ATCC, Manassas, VA, USA) were propagated in RPMI medium (Gibco, Waltham, Massachusetts, USA) containing 50 μM β-mercaptoethanol (Gibco), Glutamax 2 mM (Gibco), sodium pyruvate 1 mM (Gibco), HEPES 10 mM (Gibco), gentamicin 50 μg/mL (Sanofi-Aventis) and 10% fetal bovine serum (Sigma-Aldrich) at 37 °C in a humidified 5% CO_2_ atmosphere. For the phospho-specific flow cytometry, the THP1 cells (1 ×10^6^ cells/mL) were treated with insulin (10 nM) for 15, 30, or 60 min. For immunohistochemistry, THP1 (TIB-202, ATCC, Manassas, VA, USA) was seeded 10^6^/mL on glass chamber slides (ThermoScientific) precoated with poly-L-lysine (Sigma-Aldrich). Cells were treated with insulin 0 nM, 10 nM and 25 nM (Humalog 100 U/mL, Eli Lilly) or HDACi valproate, 50 µg/mL and 100 µg/mL, for 24 h.

### Immunophenotype characterization by flow cytometry

At the end of incubation, cells were carefully collected including detachment step with 1 mM ice cold EDTA for 15 minutes. Cells were suspended in FACS buffer (phosphate buffered saline containing 2% fetal calf serum, 0.1% sodium azide and 1 mM EDTA). Non-specific binding was blocked with human γ-globulin (Beriglobin, CSL Behring, Melbourne, Australia). Thereafter, monoclonal mouse anti-human antibodies CD4-Brilliant Violet 510 (Clone SK3, Beckton Dickinson, Franklin Lakes, NJ, USA), CD14-PE (Clone M5E2, BD), CD45RO-APC (clone UCHL1, BD), CD27-FITC (clone L128, BD) and PD1-Brilliant Violet 421 (clone EH12.1, BD) were added. All antibodies were diluted in FACS buffer to optimal concentrations.

For staining of pAKT1, cells were fixed at harvest by addition of 2× culture volume of 4% buffered formalin and incubated at 37 °C for 10 min. Cells were washed with ice-cold PBS and immediately permeabilized with ice-cold methanol for 10 min. After another wash the cells were stained with monoclonal rabbit p^S473^-AKT1 antibodies (Clone D9E, Cell Signaling Technology, Danvers, MA, USA) in dilution 1/500 in FACS buffer for 2 h at room temperature followed by secondary Alexa-fluor AF488 conjugated goat-anti-rabbit antibodies (A11034, Invitrogen) in dilution 1/200, for 1 h at room temperature.

For the histone acetylation staining, cells were permeabilized with eBioscience transcription factor staining buffer set (Invitrogen, Waltham MA, USA) and washed with the wash buffer included in the kit. Cells were incubated with rabbit anti-H3K27ac antibodies (C15410196, Diagenode, Denville, NJ, USA) in dilution 1/5000, at 4 °C over night; followed by secondary Alexa-fluor AF647 conjugated donkey-anti-rabbit antibodies (A31573, Invitrogen), in dilution 1/200, for 1 h at room temperature. For intracellular staining, rabbit gamma globulin (Jackson ImmunoResearch Laboratories, West Grove, PA, USA) was used as negative isotype control to set background signals.

The stained cells were collected 100,000 events/sample in the Cytoflex (Beckman-Coulter, Brea, CA, USA) and analyzed with Flow-Jo software version 10.10 (Ashland, OR, USA). Cell populations were gated using fluorochrome minus one staining controls. Results are presented as the gated population frequency and by mean fluorescence intensity (MFI).

### Cytokine measurements

Cytokine levels were measured by sandwich ELISAs for IFNγ (DY285B), IL-4 (DY204), and IL13 (DY213) (R&D Systems, Minneapolis, MN, USA), according to the manufacturer’s instructions.

### Transcriptional sequencing (RNA-seq)

RNA of CD4^+^ cells was prepared using the Norgen Total RNA kit (17200 Norgen Biotek, Ontario, Canada). Quality control was done by Bioanalyzer RNA6000 Pico on Agilent2100 (Agilent, Santa Clara, CA, USA). Deep sequencing was done by RNA-seq (Hiseq2000, Illumina) at the core facility for Bioinformatics and Expression Analysis (Karolinska Institute, Huddinge, Sweden). Raw sequence data were obtained in Bcl-files and converted into fastq text format using the bcl2fastq program from Illumina.

### Chromatin immunoprecipitation and sequencing (ChIP-seq)

For ChIP-seq analysis, three independent CD4^+^ cell cultures were stimulated with ConA and LPS for 24 h. The cells were cross-linked and lysed with the MAGnify Chromatin Immunoprecipitation System (492024, Applied Biosystems, ThermoFisher Scientific). After sonication, cellular debris was removed, and DNA material was pooled. After preclearing, 1% of the sample was saved as an input fraction and used as background binding. Pre-cleared chromatin was incubated with 2 μg of anti-survivin (ab192675, Abcam, Cambridge, UK), or anti-H3K27ac (C15410196, Diagenode). The immune complexes were washed, the cross-links were reversed, and the DNA was purified as recommended. The quality of purified DNA was assessed with TapeStation (Agilent, Santa Clara, CA, USA). DNA libraries were prepared with ThruPLEX (Rubicon) and sequenced at BEA with a Hiseq2000 sequencing system (Illumina). Bcl-files were converted and demultiplexed to fastq with bcl2fastq (Illumina).

### Immunohistochemistry

At harvest, cells were fixed with 4% buffered formalin for 10 min, thereafter, blocked and permeabilized for 3 h with 3% normal goat serum and 1% Triton-X100. Primary antibodies against H3K27ac (C15410196, Diagenode) and rabbit gamma globulins (Jackson ImmunoResearch Laboratories, West Grove, PA, USA) as negative control were diluted in blocking buffer and the slides were incubated over night at 4 °C. This was followed by Alexa-fluor conjugated secondary antibody donkey-anti-rabbit AF647 (Invitrogen, A31573) for 2 hours at room temperature. Autofluorescence was blocked with 0.5% Sudan Black B (Sigma-Aldrich) in 70% ethanol for 20 min at room temperature. Nuclei were stained with Hoechst 34580 (NucBlue Live Cell Stain; ThermoFisher) for 20 min and mounted with ProLong Gold antifading mounting reagent (Invitrogen).

### Confocal imaging and analysis

Fluorescence microscopy was performed using the confocal imaging system Leica SP8 (Leica Microsystems, Wetzlar, Germany) with sequential acquisition using a 40× oil objective. For the statistical analysis the images were acquired at high resolution by adding 1.5× digital zoom, which allowed viewing 20–40 nuclei per image. Within each sample, H3K27ac-positive foci were enumerated in 2–3 images resulting in 44-63 nuclei per treatment. Images were analyzed with ImageJ version 2.9 within 8-bit composite images [43]. The threshold was adjusted to optimize identification of positive spots. The nuclear area was defined by thresholding the Hoechst (blue) image and exporting the results to ROI. Enumeration of positive foci per nucleus was done using the ImageJ function FindMaxxima.

### Bioinformatics analysis

#### RNA-seq analysis

Mapping of transcripts was done using Genome UCSC annotation set for hg38 human genome assembly. Analysis was performed using the core Bioconductor packages in R-studio v. 4.4.1. Differentially expressed genes (DEG) between the samples were identified using DESeq2 [44] (v.1.44.0) with Benjamini–Hochberg adjustment for multiple testing.

#### ChIP-seq analysis

The fastq sequencing files were mapped to the human reference genome (hg38) using the STAR aligner [45] with default parameters apart from setting the alignIntronMax flag to 1 for end-to-end mapping. Quality control of the sequenced material was performed by FastQC tool using MultiQC v.0.9dev0 (Babraham Institute, Cambridge, U.K.). Peak calling was performed using the HOMER (49) findPeaks command, with 1 tag per base pair counted (-tbp 1). For peak calling in histone ChIP-seq, the option -style histone was used to find broad regions of enrichment. Peaks were filtered for the histone H3 antibody or survivin antibody IP fraction and unprocessed DNA (Input), which is a generally accepted normalization approach to identify protein-specific enrichment of DNA interaction areas [46]. A set of peaks with enrichment versus surrounding region and Input (adjusted *p* < 1e−6) was identified and quantified separately for each sample. Peaks were annotated with HOMER software in standard mode to the closest TSS. Peaks with overlapping localization by at least one nucleotide were merged and further on referred to as one peak. To quantify strength of binding and maintain consistency of comparison in the histone H3 samples and survivin sample, peak score was calculated by the position adjusted reads from initial peak region.

#### Identification of peak colocalization

The R package ChIPpeakAnno [47], version 3.38.1 was used to identify colocalization of the control ChIP-seq peaks of survivin and the histone H3K27ac peaks. The function ‘findOverlapsofPeaks’ was used, with parameters restricting the maximum gap between peak ranges to zero, indicating a minimum of one bp overlap, and connected peak ranges within multiple groups as ‘merged’. The resulting set of chromatin regions represented the genome locations possessing concomitant deposition of survivin and H3K27ac.

#### Genomic regulatory element colocalization and overlap with TF target genes

Experimentally confirmed candidate regulatory elements (RE) was obtained through the GeneHancer database version 5.9 by request [21]. The BED files of RE were combined with BED files of gene bodies and 2 kb upstream promoters of hg38 to obtain an integrated list of genomic regulatory elements. Using the ChIPpeakAnno parameters mentioned above, chromatin regions possessing colocalized survivin and H3K27ac were overlapped with the genomic regulatory elements. Further, the entire list of the genes connected to the genomic regulatory elements was retrieved used for further downstream analysis.

### Genes relating to metabolic pathways

For analysis of metabolic activity, we used the Gene Ontology database [48] to retrieve the genes annotated to the four metabolic pathways: Glycolysis, GO:0006096; Pentose Phosphate Pathway (PPP), GO:0006098; Tricarboxylic Acid (TCA) cycle, GO:0006099; and Oxidative phosphorylation (OxPhos), GO:0022900 and GO:0006119. To analyze histone acetylation activity, we retrieved the transcription levels of the genes annotated to histone acetyl transferase enzymes (HATs, GO:0000123), and genes related to histone deacetylases (HDACs), GO:0000118.

### Single cell transcriptome (scRNAseq) analysis

To analyze the metabolic activity and insulin-sensitive effects obtained through bulk transcriptomics, we used the single cell transcriptome datasets of synovial tissue [49], synovial fluid and PBMC of RA patients (*n* = 8) [50], T2D (*n* = 7) and healthy controls (*n* = 3) [23]. Using the cluster annotation of RA synovia, we mapped (using FindTransferAnchors and the MapQuery functions in Seurat) and annotated cell labels in the RA PBMC single cell dataset after merging of the PBMC samples and similar pre-processing steps as for the RA synovia. In the same manner, T cell clusters were mapped to the single cell transcriptome of PBMC from type 2 diabetes (T2D) and healthy controls. Cell type labels were transferred with good prediction scores (mean prediction score: 0.42 for PB-T2D; 0.37 for PB-RA and SF-RA) (Supplementary Fig. S3C, D). Cells were classified as high and low insulin signaling based on the mean expression of insulin signaling genes (*INSR*, *IGF1R*, *IRS1*, *IRS2*) per cell. To assess metabolic activity in the clusters, similar calculations were performed using the genes in the metabolic pathways, HATs, HDACs, and the markers of the clusters.

### Data analysis and visualization

Statistical analysis was performed using R-studio (version 4.4.1). Heatmaps were visualized using the R package ComplexHeatmap [51] version 2.20.0. Analysis for the enriched biological pathways was done through the Gene Set Enrichment Analysis (GSEA) online tool from Broad Institute, UC San Diego [52, 53].

Supplementary information is available at Cell Death & Disease’s website.

## Supplementary information

Supplementary Figure S1

Supplementary Figure S2

Supplementary Figure S3

Supplementary Figure Legends

Supplementary Table ST1

Supplementary Table ST1.1

Supplementary Table ST2

Supplementary Table ST3

Supplementary Table ST4

Supplementary Table ST5

Supplementary Table ST6

Supplementary Table ST7

## Data Availability

The biological material used in this study is summarized in Supplementary Table ST1. ChIPseq datasets for CD4^+^ samples immunoprecipitated with survivin and H3K27ac are available at Gene Expression Omnibus (GEO) of the National Center for Biotechnology Information (NCBI) with accession GSE282301. Transcriptome sequencing data of insulin-stimulated CD4^+^ cells are deposited in NCBI GEO with accession GSE282515. Transcriptome sequencing data of CD4^+^ cells from RA patients and healthy controls are deposited in NCBI GEO with accession GSE282517. Transcriptome sequencing data of HDACi-treated CD4^+^ cells are available at NCBI GEO, accession GSE132053 [54]. Single-cell sequencing data for CD4^+^ cells from blood and synovial fluid [50], RA synovial tissue [49] are publicly available. Single cell sequencing data for PBMCs of T2D and HC are accessible in NCBI GEO with accession GSE165816 [23]. For assessment of effect of metformin on the transcriptome of PBMC, we used paired transcriptome data from GSE153792 [24]. For assessment of the effect of anti-rheumatic drugs on transcriptome of CD4^+^ cells, we used paired transcriptome data of CD4^+^ cells isolated from untreated RA patients (*n* = 9) and treated with JAKi (*n* = 25) [12], CTLA4 fusion protein abatacept (*n* = 22 [28]), folic acid antagonist methotrexate (*n* = 28 [27]) and IL6 receptor inhibitor tocilizumab (*n* = 6 [29]).
