# Supplementary figures and images for: Insulin enables acquisition of the IL7R+ memory phenotype in PD1+ T cells in RA tissues
Source: Cell Death Dis. 2026 May 25;17(1):506. doi: 10.1038/s41419-026-08916-6 (PMC13201554; doi:10.1038/s41419-026-08916-6)

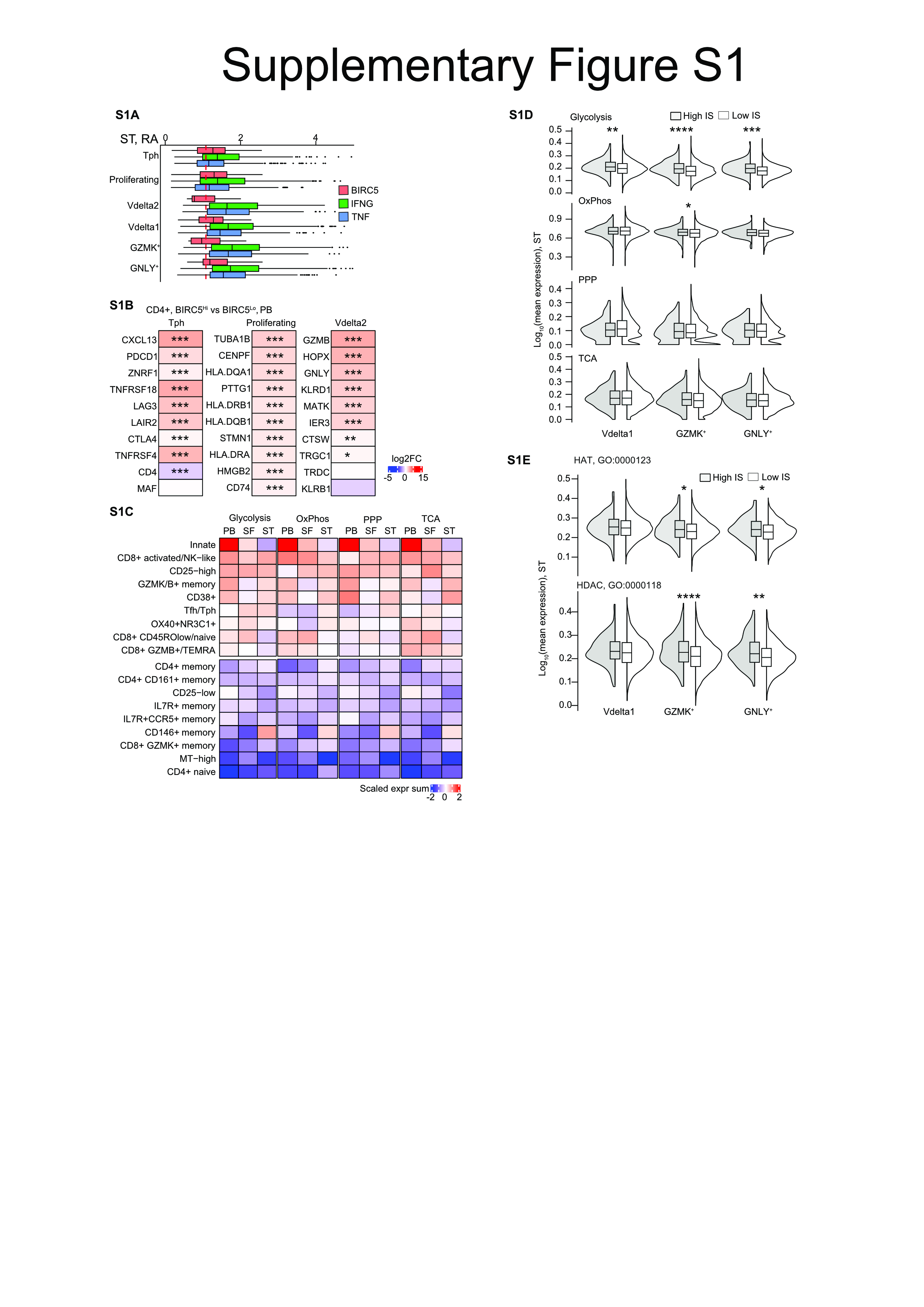

Supplement: Supplementary file 1 — Supplementary Figure S1 [file 41419_2026_8916_MOESM1_ESM.tif]

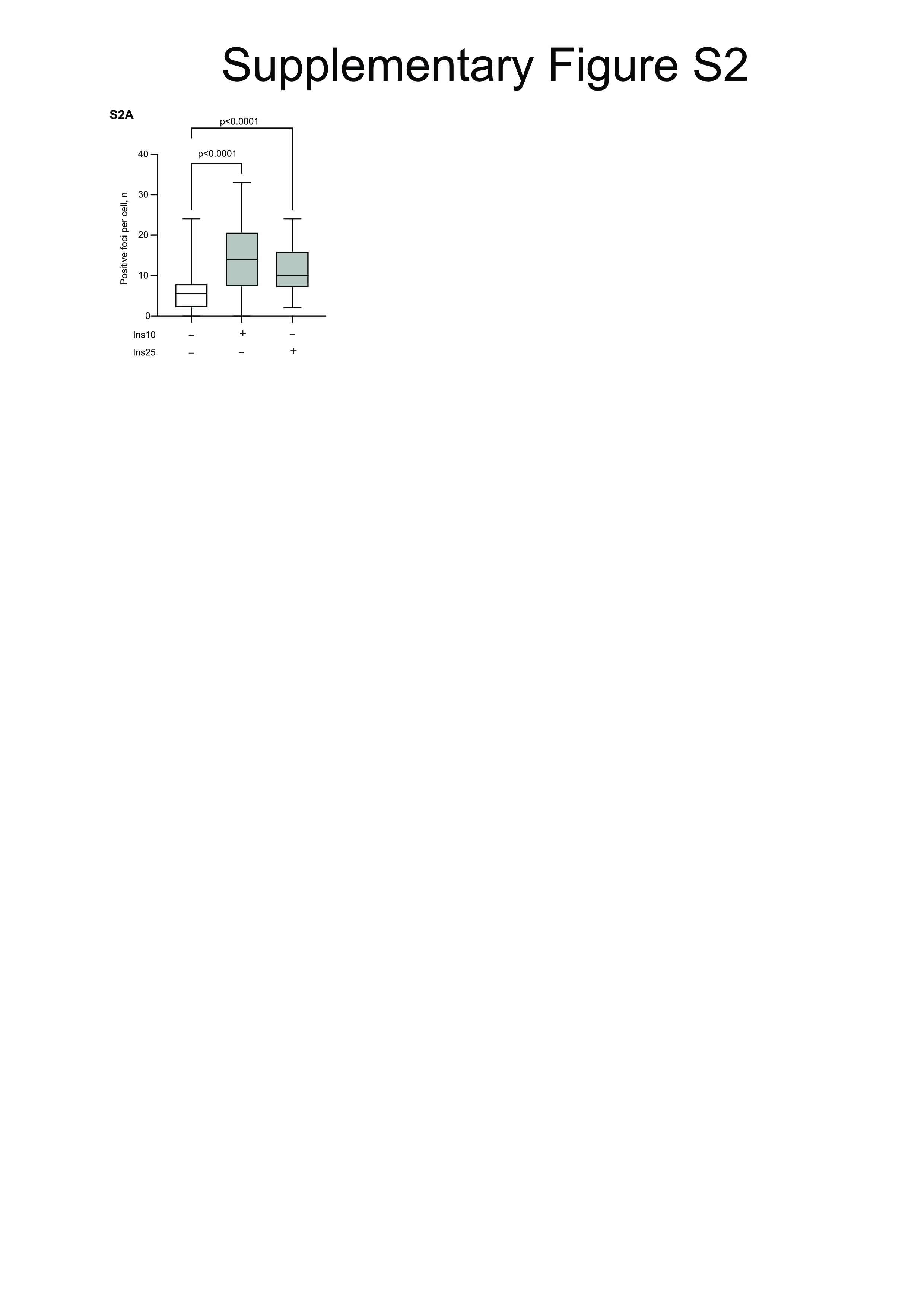

Supplement: Supplementary file 2 — Supplementary Figure S2 [file 41419_2026_8916_MOESM2_ESM.tif]

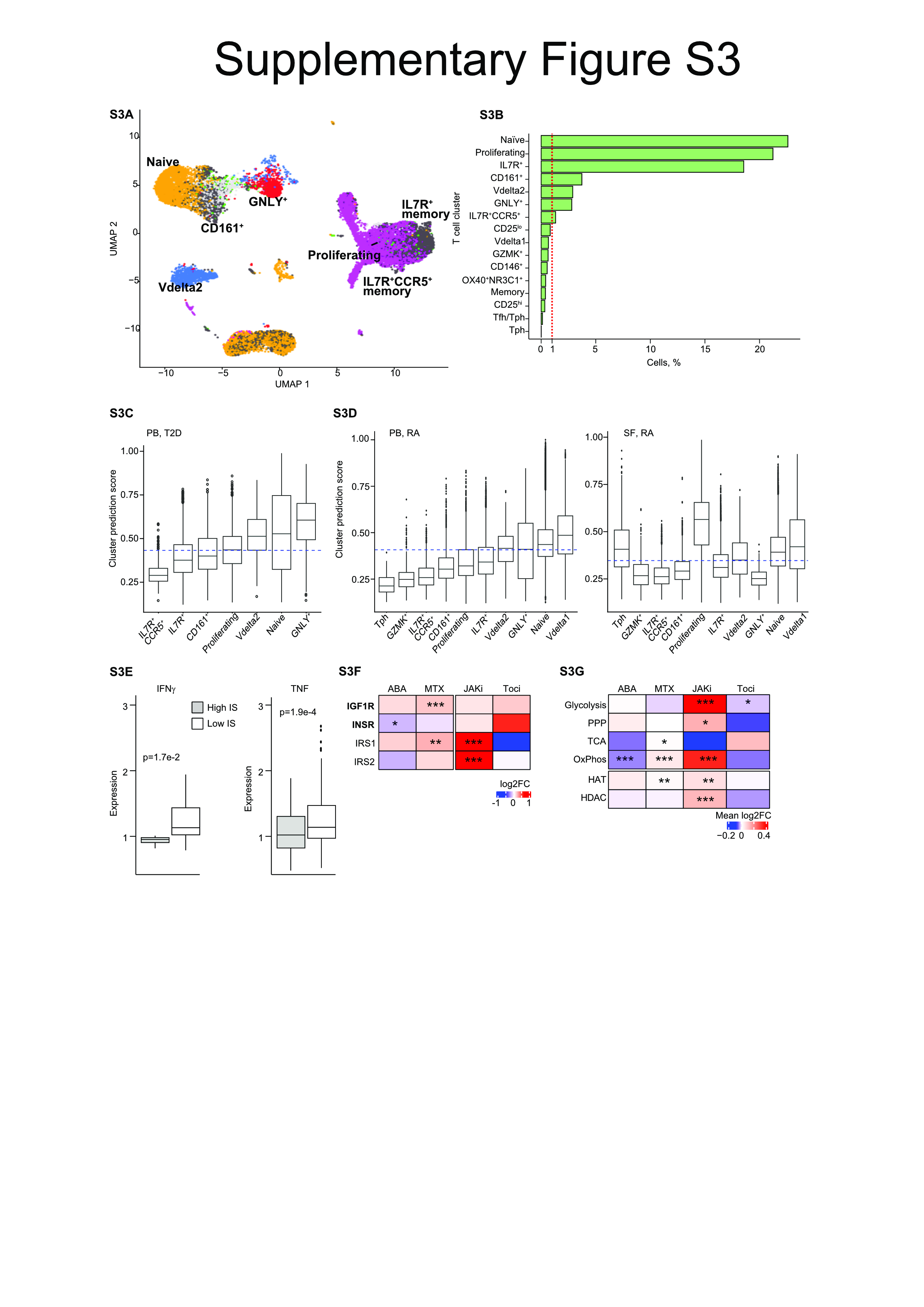

Supplement: Supplementary file 3 — Supplementary Figure S3 [file 41419_2026_8916_MOESM3_ESM.tif]
