# Supplementary material for: Insulin enables acquisition of the IL7R+ memory phenotype in PD1+ T cells in RA tissues
Source: Cell Death Dis. 2026 May 25;17(1):506. doi: 10.1038/s41419-026-08916-6 (PMC13201554; doi:10.1038/s41419-026-08916-6)
Supplement: Supplementary file 4 — Supplementary Figure Legends [file 41419_2026_8916_MOESM4_ESM.docx]

# Supplementary Figure legends

**Supplementary Figure S1**

S1A. Box plot of gene expression in CD4^+^ T cells clusters of RA synovial tissue (ST), by single cell RNA-seq.

S1B. Heatmap of expression difference of the T cell cluster markers in blood CD4^+^ cells split by high and low BIRC5 expression, by RNA-seq. Expression difference is calculated by DESeq2 analysis as log2FoldChange (FC). Asterisks indicate nominal p-values * < 0.05, ** < 0.01, *** <0.001, **** <0.0001

S1C. Heatmap of standardized expression sum of the metabolic pathway genes in peripheral blood (PB), synovial fluid (SF), and synovial tissue (ST) of RA patients, by scRNA-seq. GO-terms used for pathway annotation are glycolysis (GO:0006096); oxidative phosphorylation (OxPhos, GO:0006119 and GO:0022900); pentose phosphate pathway (PPP, GO:0006098); tricarboxylic acid cycle (TCA, GO:0006099). Metabolically active T cell clusters are red and inactive clusters are blue

S1D. Violin and box plot of mean expression of metabolic pathways, by RNA-seq, in high and low insulin signaling (IS) cells in metabolically inactive *BIRC5*^Hi^CD4­^+^ clusters. GO-terms used for pathway annotation are glycolysis (GO:0006096); oxidative phosphorylation (OxPhos, GO:0006119 and GO:0022900); pentose phosphate pathway (PPP, GO:0006098); tricarboxylic acid cycle (TCA, GO:0006099).

S1E. Violin and box plot of mean expression of HAT and HDAC enzymes in metabolically inactive *BIRC5*^Hi^CD4^+^ clusters. GO-terms used for annotation HAT (GO:0000123) and HDAC (GO:0000118). P-values are calculated by Wilcoxon unpaired test. Asterisks indicate p-values * < 0.05, ** < 0.01, *** <0.001, **** <0.0001

**Supplementary Figure S2**

S2A. Box plot of H3K27ac-positive foci number per nucleus in THP1 cultures stimulated with insulin. P-values are calculated by Wilcoxon unpaired test.

**Supplementary Figure S3**

S3A. UMAP plot of T cell cluster mapped to single cell transcriptome of PBMCs from type 2 diabetes patients and healthy controls.

S3B. Bar plot showing percentage of cells in each T cell cluster in single cell transcriptome of PBMCs from type 2 diabetes patients and healthy controls.

S3C. Box plot of cluster prediction score in single-cell RNA-seq of T cells in peripheral blood of T2D patients after mapping from T cell clusters from synovial tissue of RA patients. The blue dotted line represents the mean prediction score of all clusters.

S3D. Box plot of cluster prediction score in single-cell RNA-seq of T cells in peripheral blood (PB) and synovial fluid (SF) of RA patients after mapping from T cell clusters from synovial tissue of RA patients. The blue dotted line represents the mean prediction score of all clusters.

S3E. Box plot of IFNG, and TNF expression in cells with high and low insulin signaling in IL7R+ cluster in PBMC of T2D patients. P-values are calculated by Wilcoxon unpaired test.

S3F. Heatmap of expression difference sum, by RNA-seq, in insulin signaling (E2) in CD4+ T cells of RA patients treated with abatacept (ABA, n=22, paired), methotrexate (MTX, n=28, paired), JAKi (35 treated, 5 untreated), and tocilizumab (TOCI, n=6, paired). Genes connected to cis-RE containing survivin-H3K27ac co-deposition are indicated bold. P-values are calculated by DESeq2 analysis. Asterisks indicate p-values * < 0.05, ** < 0.01, *** <0.001, **** <0.0001

S3G. Heatmap of expression difference sum, by RNA-seq, in metabolic pathways in in CD4^+^ T cells of RA patients treated with abatacept (ABA, n=22, paired), methotrexate (MTX, n=28, paired), JAKi (35 treated, 5 untreated), and tocilizumab (TOCI, n=6, paired). Difference in expression is calculated by DESeq2 analysis and presented by log2 fold change (FC). Asterisks indicate nominal p-values, *<0.05, ** < 0.01, *** < 0.0001
